# Polarization‐Controlled Wavelength‐Tunable Pulsed Laser Based on Metafiber

**DOI:** 10.1002/nap2.70278

**Published:** 2026-08-28

**Authors:** Chenxi Zhang, Zanzhu Pan, Chenlu Zhao, Ziyu Gu, Xiuhan Jing, Wenhao Lyu, Jiale Chao, Bo Fu

**Affiliations:** ^1^ Hangzhou International Innovation Institute Beihang University Hangzhou China; ^2^ Key Laboratory of Precision Opto‐Mechatronics Technology of Education Ministry School of Instrumentation and Optoelectronic Engineering Beihang University Beijing China; ^3^ National Superior College for Engineers Beihang University Beijing China

**Keywords:** metafiber, polarization control, Q‐switched pulse, wavelength‐tunable pulsed laser

## Abstract

Wavelength‐tunable pulsed lasers have the ability to flexibly adjust laser energy and wavelength according to the properties of different materials platforms and experimental requirements, thereby markedly enhancing efficiency and precision. Thus, wavelength‐tunable pulsed lasers have attracted significant attention in fields such as wavelength division multiplexing, precision material processing, and spectroscopic analysis. Here, a polarization‐sensitive Au‐nanorod metasurface has been proposed that integrates saturable absorption with wavelength‐tuning capabilities. By fabricating this metasurface structure on the facet of the fiber, known as a metafiber, low‐loss coupling between the metasurface and the fiber laser system is achieved. In experiments, we have successfully demonstrated Q‐switched pulse, achieving precise control over pulse width (13.5–29.8 μs), repetition rate (15.3–43.7 kHz), and pulse energy (15.2–30.7 nJ) through meticulous adjustment of the pump power. Notably, by modulating the input polarization state, the response wavelength of the metafiber can be effectively controlled, enabling wavelength‐tunable pulsed laser output within a 5‐nm range. This achievement introduces a new concept and provides a viable solution for the design and development of next‐generation compact, high‐efficiency, and multifunctional laser systems.

## Graphical abstract

A polarization‐sensitive Au‐nanorod metasurface has been proposed that integrates saturable absorption with wavelength‐tuning capabilities. By fabricating this metasurface structure on the facet of the fiber, known as a metafiber, low‐loss coupling between the metasurface and the fiber laser system is achieved. Notably, by modulating the input polarization state, the response wavelength of the metafiber can be effectively controlled.

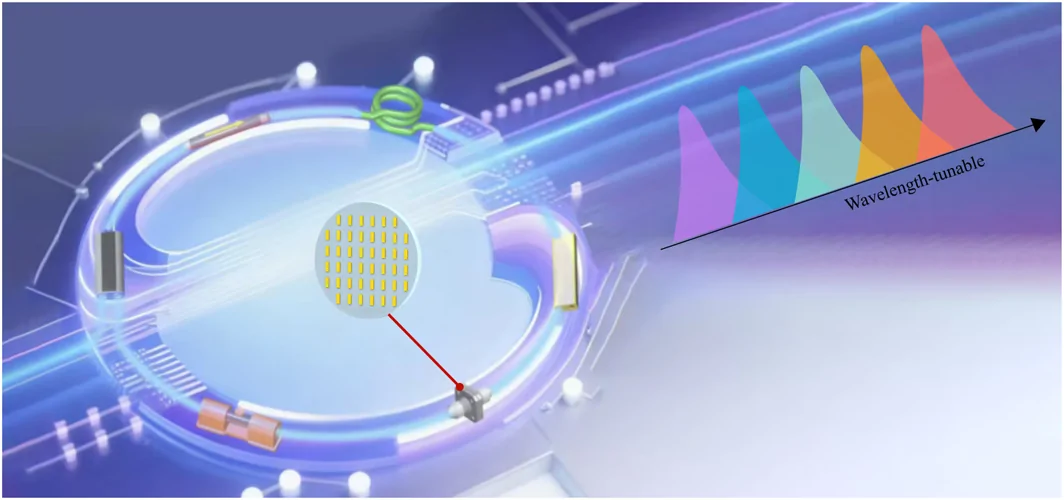

## 1 Introduction

Pulsed lasers are widely used in various fields such as material processing [1], medical care [2, 3], and scientific research [4] due to their high energy density and high resolution. Among them, wavelength‐tunable pulsed lasers have garnered significant attention. Their capacity to flexibly adjust laser energy and wavelength in accordance with varying material properties and experimental requirements markedly enhances efficiency and precision [5, 6]. Consequently, wavelength‐tunable pulsed lasers have become exceedingly valuable in applications such as precision biomedical imaging [7], medical diagnostics [8], and spectroscopic analysis [9] where the ability to tailor laser parameters is crucial for optimal performance and results. In recent years, the concept of metasurfaces has emerged with the development of advanced photonic materials and nanostructures [10, 11]. Metasurface, as a general term for ultrathin nanoscale materials with thicknesses smaller than the optical wavelength, is typically composed of periodic or quasi‐periodic subwavelength unit‐cell structures [12, 13]. Precisely designed micro‐ and nanostructures enable them to manipulate the phase, amplitude, polarization, and other characteristics of electromagnetic waves [14, 15], allowing for flexible control of light [16, 17, 18, 19]. Notably, polarization‐sensitive metasurfaces have demonstrated remarkable potential in a variety of applications including polarization conversion, polarization‐dependent imaging, and optical communications [20, 21].

Despite the significant progress achieved in wavelength‐tunable pulsed lasers, existing technologies still encounter numerous challenges [9]. Traditional wavelength‐tuning methods, such as fiber filter and intracavity gratings [6, 17, 18, 19], often face limitations in terms of tuning range, tuning speed, and stability. Moreover, integrating these tuning mechanisms into compact and efficient laser systems remains a significant challenge. On the other hand, although the ability of metasurfaces to manipulate light at the nanoscale has been extensively investigated [22, 23], their application in laser systems, particularly for wavelength‐tunable pulsed lasers, is still in its infancy. In recent years, researchers have begun to explore the application of metasurfaces in laser systems, such as beam shaping, frequency conversion, and nonlinear optics [12]. However, research on combining metasurfaces with pulse generation mechanisms to achieve wavelength‐tunable pulsed lasers is still relatively limited. Q‐switching technology is a technique employed to generate high‐power pulsed lasers by modulating the loss within the laser cavity, thereby controlling the energy storage and release process [24, 25]. Integrating metasurfaces as both pulse generation and wavelength‐tuning devices still poses certain challenges, yet it holds the promise of developing a new generation of compact, efficient, and multifunctional laser systems.

In this work, we propose a metafiber based on a polarization‐sensitive nanorod metasurface structure. The metafiber can generate Q‐switched pulses as saturable absorber (SA) while achieving wavelength‐tunable pulsed laser emission through polarization control, as illustrated in Figure 1. The metasurface is integrated onto the facet of a single‐mode fiber to form the metafiber, ensuring low insertion loss between the metasurface structure and the laser system. Under a pump power of 50 mW, the metafiber generates Q‐switched laser pulses with a center wavelength of 1558.6 nm, a pulse width of 29.4 μs, and a repetition rate of 13.8 kHz. By adjusting the pump power, controllable outputs of pulse width, repetition rate, and pulse energy can be realized. Subsequently, by tuning the polarization controller, wavelength‐tunable Q‐switched pulsed laser emission is further achieved. Au nanorod metafiber integrates saturable absorption, wavelength selectivity, and polarization‐dependent tunability into a single mode fiber device. In this device, the output pulse characteristics can be modulated on demand during laser generation. The key distinction with traditional SA is that our metafiber simultaneously acts as both the mode‐locking or Q‐switching element and the wavelength‐selective tuning element, eliminating the need for additional intracavity components. Our research not only contributes to a deeper understanding of light–matter interactions at the nanoscale but also holds significant implications for practical applications such as high‐precision material processing, medical diagnostics, and spectroscopic analysis.

**FIGURE 1:**
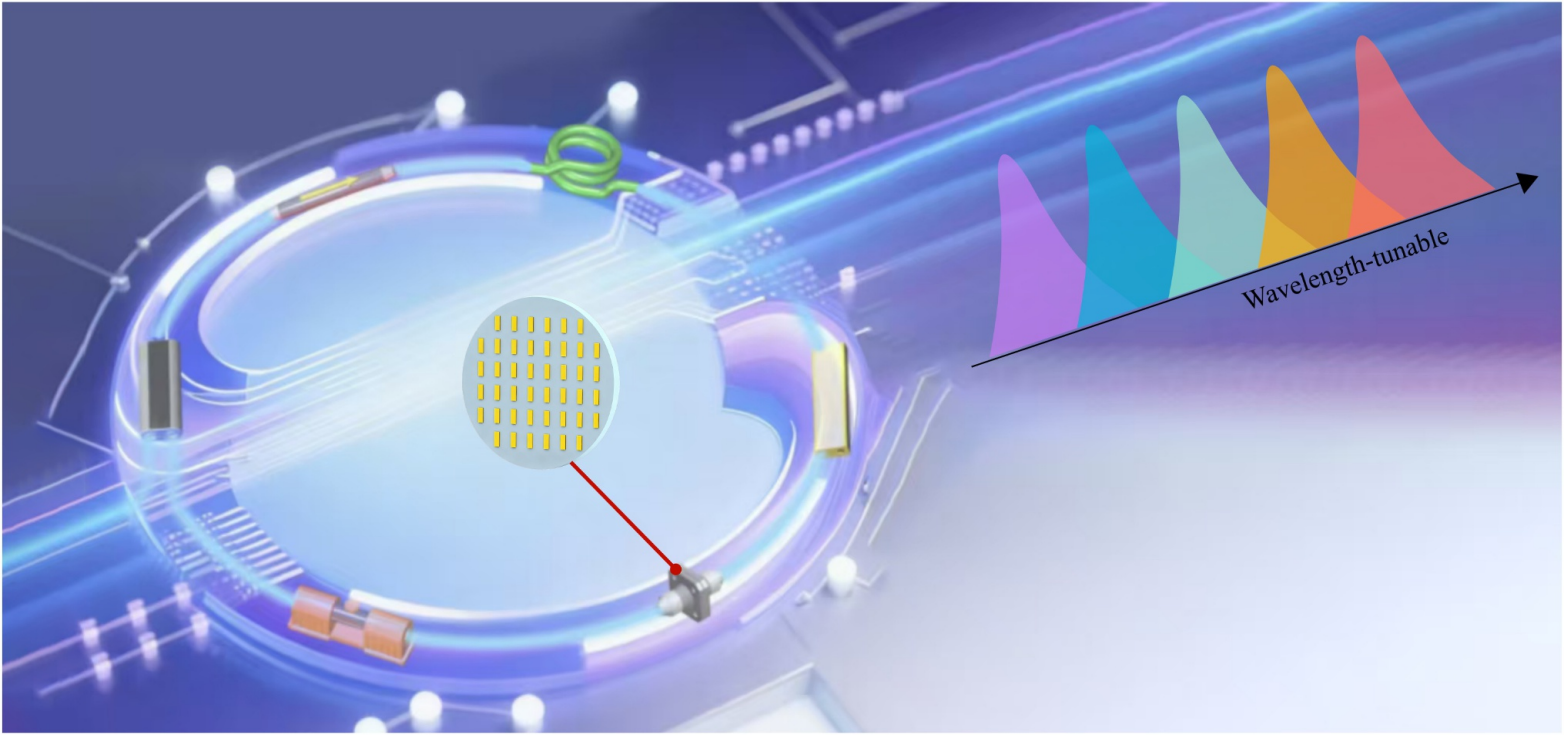
Wavelength‐tunable pulsed laser via a metafiber.

## 2 Design and Characterization of Metafibers

In order to achieve a metasurface structure with plasmonic resonance characteristics at the 1.5 μm wavelength and strong saturable absorption capabilities, an Au‐nanorod unit was designed, as illustrated in Figure 2a. The metafiber consists of an SMF and a metasurface in which the SMF exhibits strong anti‐interference characteristics. Therefore, the metasurface is the primary factor governing the stability of the metafiber. The metasurface in this work is made of Au, which exhibits excellent oxidation resistance and high thermal conductivity. The dimensions of the Au‐nanorod were precisely calculated, with a length of 470 nm, a width of 160 nm, and a height of 55 nm, as shown in Figure 2a. Based on previous research findings, this size of the Au‐nanorod structure can produce plasmonic resonance at 1575 nm [20]. Building on this foundation, the facet of fiber was utilized as the substrate, and focused ion beam etching technology was employed to precisely fabricate the structure, successfully constructing a metafiber, as shown in Figure 2b,c. The metafiber is fabricated by first depositing an Au thin film onto the fiber end face through physical vapor deposition, as illustrated in Figure 2d, focused ion beam milling (30 kV, 10 pA, Ga^+^) is employed to directly pattern the nanostructure into the film, as shown in Figure 2e. The most significant advantage of this technique is the precise patterning of nanostructures without multistep processes such as resist coating, exposure, and development. The localized surface plasmon resonance of Au‐nanorods can be precisely tuned across a broad spectral range from the visible to the near‐infrared region by varying the aspect ratio. Direct fabrication of Au‐nanorods on the fiber end face enables strong coupling with the fiber mode, yielding an intrinsic broadband optical response for wavelength tuning. Moreover, this structure couples efficiently to the fiber mode with the insertion loss below 0.5 dB. Au itself exhibits high thermal conductivity and a high damage threshold, enabling high‐power pulse generation and enhancing output stability. In addition, the Au nanorod metasurface enables precise control over dimensions and morphology. Combined with electron‐beam lithography or nanoimprint lithography, this approach allows high‐throughput and reproducible fabrication, ensuring high device‐to‐device consistency for practical applications.

**FIGURE 2:**
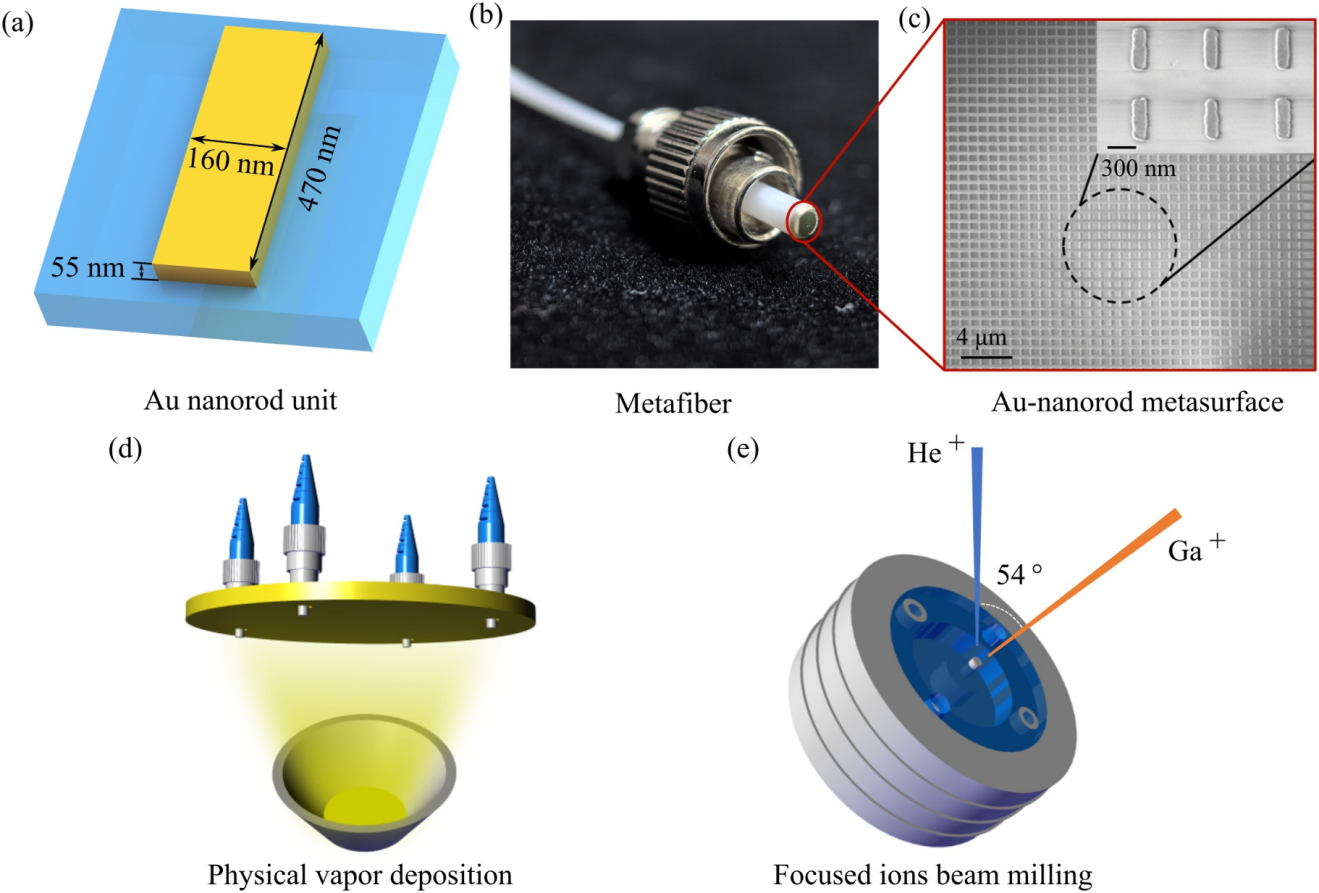
Polarization‐sensitive Au‐nanorod design and characterization. (a) Dimensions of Au‐nanorod unit. (b) Physical image of Au‐nanorod metafiber. (c) Physical image of metasurface micrograph. (d) Physical vapor deposition. (e) Focused ion beam milling.

The Au‐nanorods were arranged with a period of 750 nm and were closely integrated with the ceramic facet of the fiber during the fabrication of the metasurface structure. An extinction spectroscopy setup is designed and constructed to characterize the linear optical properties of the metafiber, as shown in Figure 3a. This setup combines free‐space and fiber‐optic components where the free‐space optical elements adjust the polarization state of the incident light. The light source is a supercontinuum source providing broadband light from 400 to 2400 nm. To prevent sample damage, the output light first passes through a neutral density filter for uniform intensity attenuation and then through a notch filter to suppress specific wavelengths. Subsequently, a polarization control module consisting of a polarizer and a half‐wave plate adjusts the polarization state of the incident light. The beam spot diameter is then adjusted using two objectives. Finally, the light beam is coupled into the SMF through a fiber collimator, and the transmitted signal is directed to a spectrometer for spectral detection. A standard SMF is used as the reference signal *T*_ref_ to calibrate the system noise and losses. Subsequently, the metafiber is connected to acquire the sample signal *T*_nr_. The extinction spectrum is thus defined as *E*_xt_ = (*T*_ref_–*T*_nr_)/*T*_ref_. The results indicate that when the polarization direction is varied from 0° to 90° in steps of 10°, the transmitted intensity at the resonant wavelength increases by 10%, as shown in Figure 3b. The plasmonic resonance peak is located at approximately 1575 nm with a full width at half maximum of 200 nm. To test the transmission characteristics of the metafiber, a nonlinear optical testing setup was constructed in this work, as shown in Figure 3c. The setup mainly includes a pulsed pump source, a variable attenuator, a 50:50 coupler, and two power meters. Experimental results show that with increasing input power, the transmission spectrum of metafiber displays typical saturable absorption characteristics, as shown in Figure 3d. Specifically, the metafiber has a modulation depth of 15.1% and a saturable absorption intensity of 0.28 MW/cm^2^.

**FIGURE 3:**
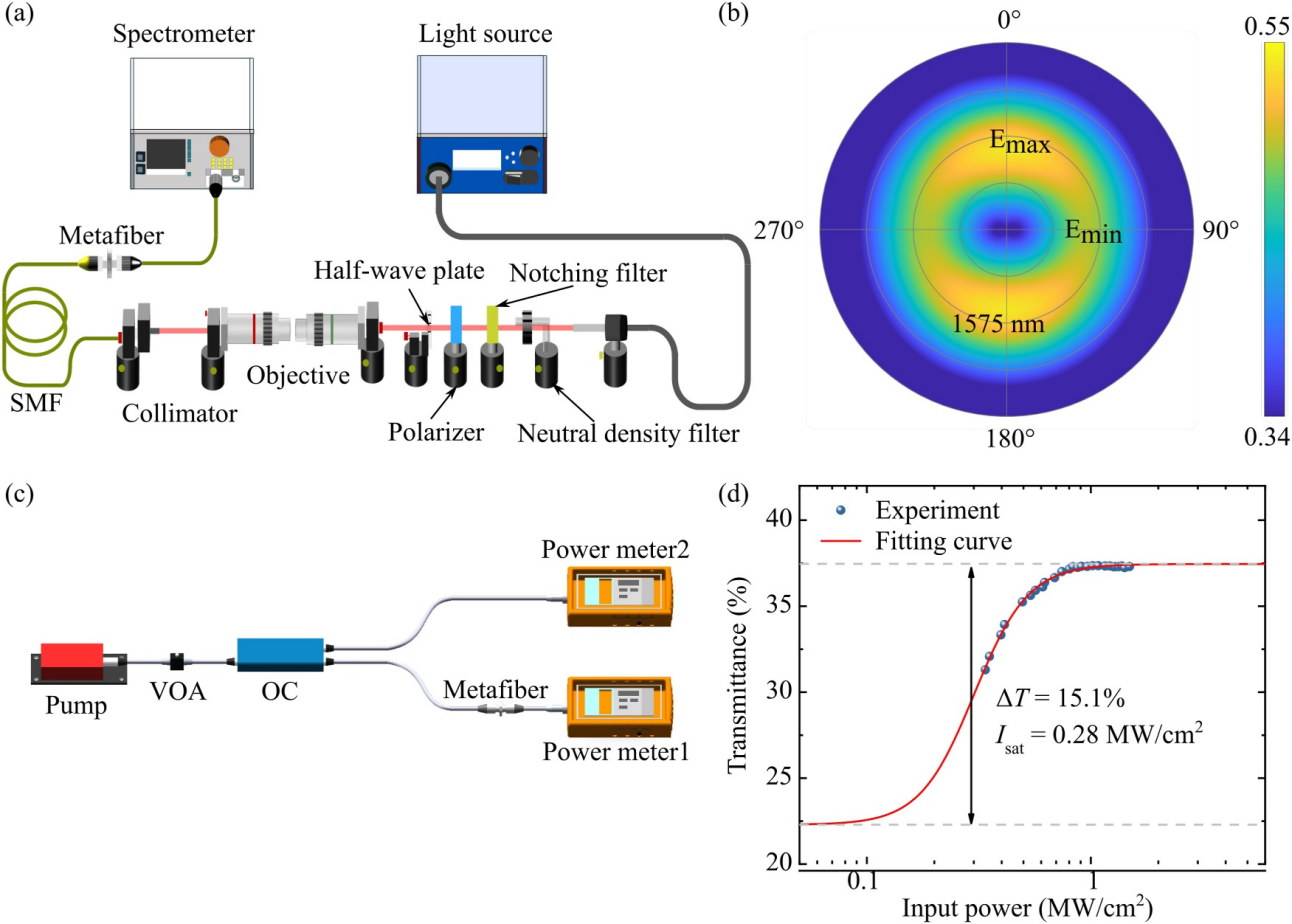
Optical characterization of metafiber. (a) Polarization‐dependent characterization testing setup for setup. (b) Extinction spectrum of metafiber. (c) Nonlinear optical transmission testing setup for metafiber. (d) Nonlinear transmission curve of metafiber.

## 3 Results and Discussion

In this work, a ring all‐fiber laser was designed and constructed [26, 27], as shown in Figure 4. The laser cavity comprises the following key components: a 980‐nm pump, a wavelength division multiplexer (WDM), an erbium‐doped fiber (EDF, 0.7 m), an optical coupler (OC), a metafiber‐SA, a polarization controller (PC), and an isolator. The 980 nm pump provides the necessary energy input to excite the erbium ions in the EDF. The WDM is used to effectively couple the 980 nm pump light and the 1550 nm signal light into the single‐mode fiber, enabling their co‐propagation. The OC has a splitting ratio of 80:20 with 20% of the light outputted from the cavity for detection and analysis of the laser characteristics, whereas the remaining 80% of the laser light is fed back into the laser to continue participating in the resonant process. The PC achieves flexible control of the polarization state within the laser cavity by precisely adjusting the angles of the three‐loop PC. Finally, the polarization‐independent isolator ensures the unidirectional transmission of the 1550 nm beam within the fiber laser, effectively preventing the interference of back‐reflected light on the laser performance.

**FIGURE 4:**
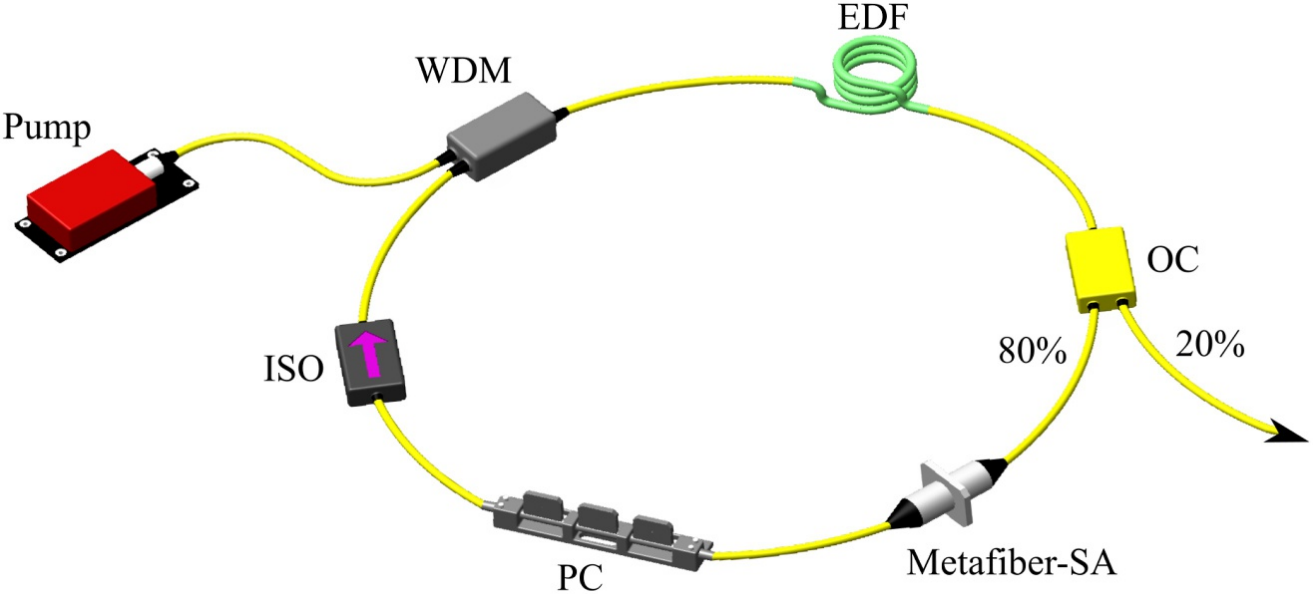
Setup of all‐fiber pulsed laser.

The generation of Q‐switched pulses is initiated when the pump power reached 50 mW. Under this condition, the laser output characteristics are shown in Figure 5. The center wavelength of the Q‐switched pulses is 1558.6 nm with the spectrum depicted in Figure 5a. The pulse train generated under these conditions exhibits remarkably stable output characteristics, which are crucial for consistent laser performance. Such stability indicates that the nonlinear modulation provided by the metafiber‐SA operates effectively within the designed cavity structure, ensuring reliable passive Q‐switching behavior. The pulse interval between successive pulses is measured to be 72.5 μs, as clearly demonstrated in Figure 5b. This pulse interval corresponds to the repetition rate of 13.8 kHz in the RF spectrum, as shown in Figure 5c, and the signal‐to‐noise ratio (SNR) at the repetition frequency is 37.6 dB. The relatively low SNR in the current experiment stems from two main sources. First, the PC sensitive to ambient temperature and mechanical vibrations, resulting in slow polarization drift. Second, the pulse‐to‐pulse timing jitter isinherently large during the buildup of passive Q‐switched pulses. The width of a single pulse is 29.4 μs, as shown in Figure 5d.

**FIGURE 5:**
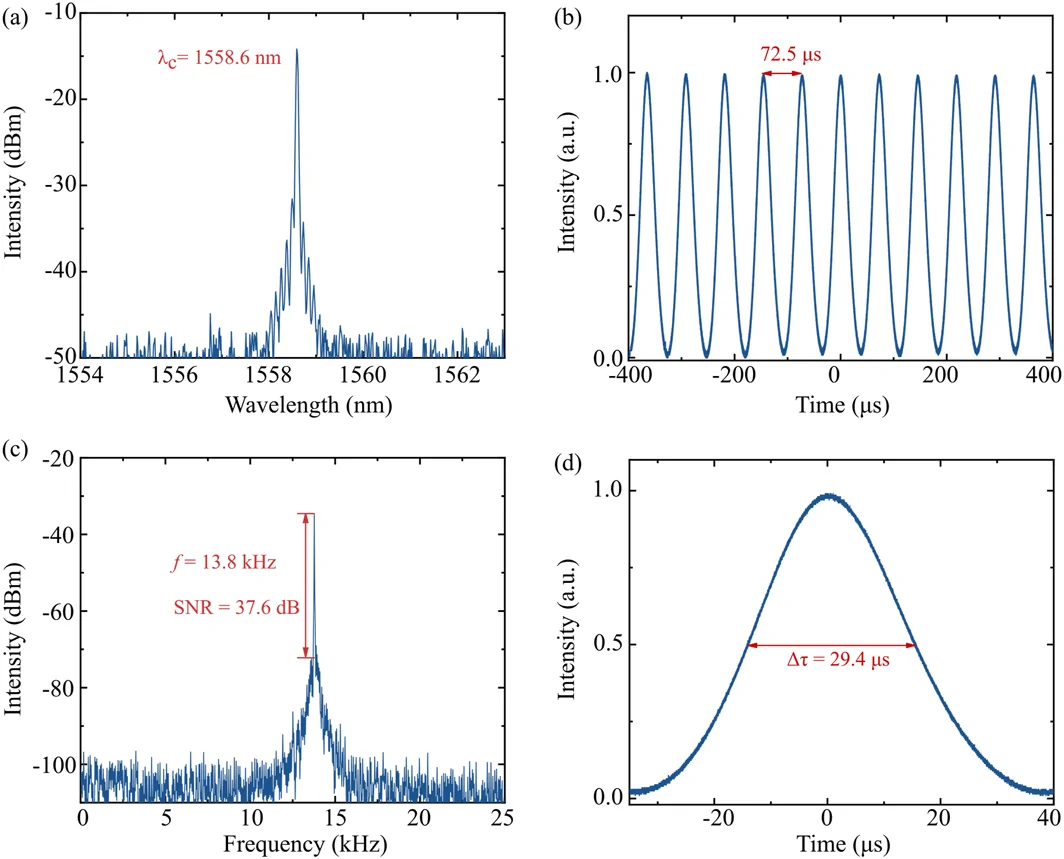
Output characteristics of Q‐switched pulsed laser based on polarization‐sensitive Au‐nanorod metafiber. (a) Spectrum of the pulsed laser. (b) Pulse train profile. (c) Frequency spectrum. (d) Single pulsed profile.

The property of the Q‐switched pulsed laser can be optimized by adjusting the pump power to achieve higher repetition frequency and narrower pulse width. Increasing the pump power can effectively enhance the gain level of the laser, thereby increasing the pulse energy and repetition rate and potentially further compressing the pulse width. However, it is important to note that increasing the pump power may affect the thermal stability of the laser. Therefore, a balance between pump power and laser property needs to be carefully considered in the experiment.

Subsequently, this work conducted a comprehensive analysis of the evolution of laser output characteristics with pump power. As illustrated in Figure 6a, the Q‐switched pulsed laser has a threshold at a pump power of 50 mW after which the output power increases linearly. With the pump power increases, the pulse train displayed on the oscilloscope, as exhibited in Figure 6b, maintains stable output with gradually decreasing pulse interval. In Figure 6c, the pulse width decreases progressively with increasing pump power, reaching a minimum width of 13.5 μs. The repetition rate increases with the pump power, reaching a maximum of 43.7 kHz. With the increase of output power and repetition rate and the decrease of pulse width, both the pulse energy and peak power are increased. The maximum pulse energy achieved is 30.7 nJ, and the maximum peak power is 2.3 mW, as depicted in Figure 6d. These results indicate that the output characteristics of the Q‐switched pulsed laser can be effectively optimized by adjusting the pump power, to achieve higher repetition rate, narrower pulse width, higher pulse energy, and peak power. However, the increase of pump power does not affect the spectral characteristics. In order to realize wavelength tunability, alternative nonlinear control methods are required.

**FIGURE 6:**
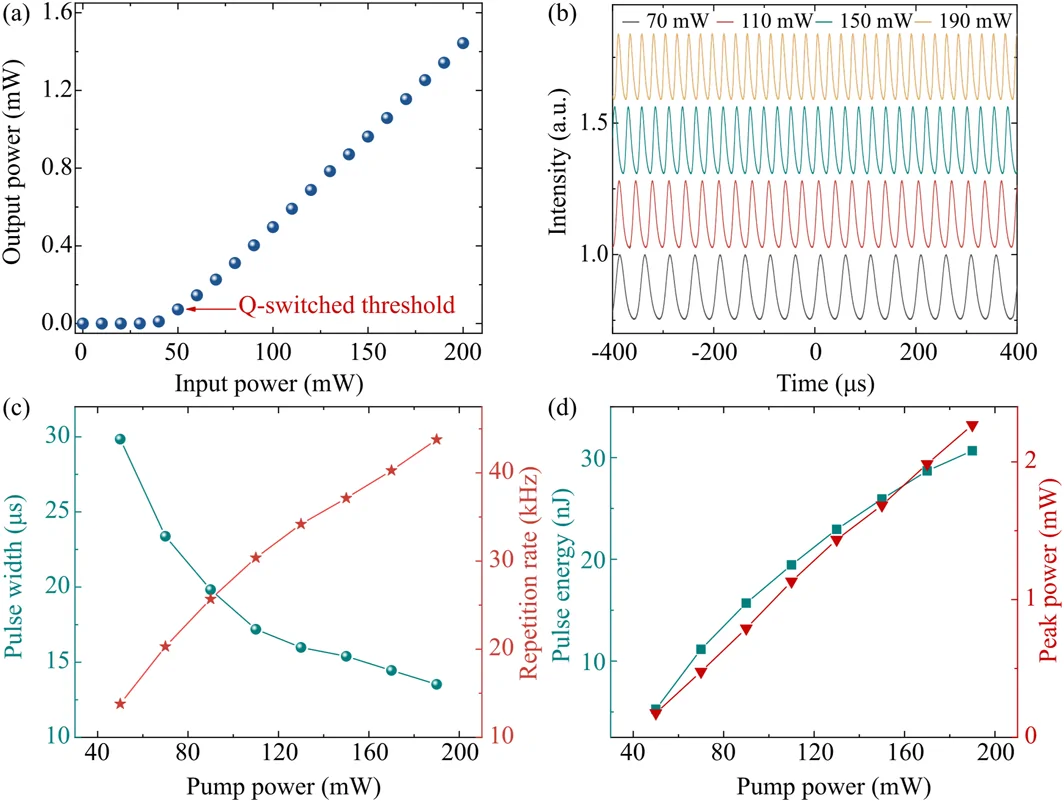
Evolution of pulsed laser output characteristics with pump power. (a) Output power curve. (b) Evolution of pulse train in oscilloscope with pump power. (c) Evolution of pulse width and repetition rate with pump power. (d) Evolution of pulse energy and peak power with pump power.

The frequency‐domain characteristics of pulsed lasers are closely related to the gain property and nonlinearity within the laser cavity [28]. In a stable laser system, the characteristics of the gain medium generally remain constant. Therefore, to achieve control over the center wavelength of the pulsed laser, nonlinear optical control methods are usually required. In this work, we propose an innovative approach using a polarization‐sensitive nanorod metasurface structure to achieve wavelength‐tunable pulses. Rotating the intracavity PC selectively excites different plasmonic resonant modes of the metafiber, enabling wavelength tuning. Thus, a wavelength‐tunable Q‐switched pulsed laser is achieved, as depicted in Figure 7a. The wavelength tuning mechanism relies on the intrinsic anisotropic plasmonic resonance of the Au‐nanorods. The geometric shape‐anisotropy of these nanorods supports a continuum of orientation‐dependent plasmonic resonances of which the longitudinal and transverse modes are the two orthogonal extremes [20]. Rotating the intracavity PC continuously adjusts the polarization state of the light, thereby exciting plasmonic modes at varying orientations across the nanorod ensemble and generating a continuous shift in the resonant wavelength. This is fundamentally different from conventional wavelength‐tuning mechanisms, such as temperature tuning, stress tuning, and birefringent filtering, which rely on external control of bulk components [29, 30, 31]. The metafiber not only exhibits saturable absorption property but also allows effective control of the resonant wavelength by adjusting the polarization state. In the experiment, with the constant pump power, the Q‐switched pulsed wavelength can be tuned over a range of 5 nm by adjusting the PC within the laser cavity as illustrated in Figure 7b. The localized surface plasmon resonance wavelength of Au‐nanorods is determined by the aspect ratio and can be tuned from approximately 600 nm to over 1000 nm by varying the synthesis conditions [32]. The material itself exhibits much broader wavelength selectivity than the demonstrated 5 nm tuning range. The main factors limiting the tuning range include the spectral dependence of cavity losses, the limited adjustment range of the PC, and mode competition [33, 34]. Cavity losses arise from metasurface scattering and polarization‐dependent insertion loss, spectrally constraining the region where round‐trip gain exceeds loss. The limited adjustment range of the PC restricts access to the full range of polarization‐dependent coupling conditions. Additionally, mode competition further narrows the region of stable single‐mode operation. Future work will focus on further optimizing the metasurface structure to expand the tuning range and improve the overall performance of the laser system.

**FIGURE 7:**
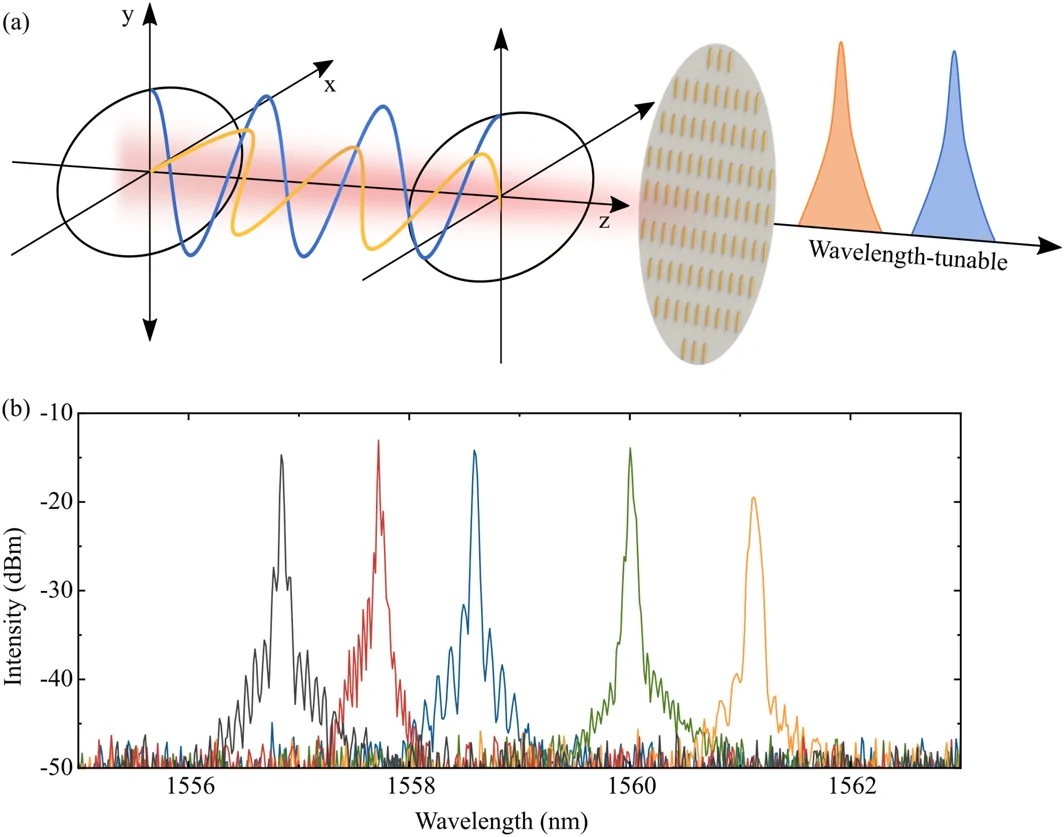
Wavelength‐tunable pulsed laser based on polarization‐sensitive metafiber. (a) Mechanism of wavelength tunability. (b) Spectra of wavelength‐tunable laser.

## 4 Conclusion

In conclusion, we propose a nonlinear optical device, metafiber that combines a SA with wavelength tunability, effectively integrating a metasurface for optical modulation with a low‐loss fiber jumper. An Au‐nanorod metasurface that is directly integrated onto the fiber end facet for low‐loss insert to the fiber laser. In the experiment, we successfully realize Q‐switched pulse generation and the controllable adjustment of the repetition rate, pulse width, pulse energy, and peak power through the regulation of the pump power. Moreover, according to the polarization‐sensitive properties of the Au‐nanorod metasurface structure, we successfully achieve the wavelength‐tunable pulsed laser with the range of 5 nm.

## Author Contributions

**Chenxi Zhang:** writing – original draft, investigation, data curation, conceptualization, project administration, writing – review and editing, methodology. **Zanzhu Pan:** writing – original draft, writing – review and editing. **Chenlu Zhao:** writing – original draft, writing – review and editing. **Ziyu Gu:** writing – original draft, writing – review and editing. **Xiuhan Jing:** writing – review and editing, writing – original draft. **Wenhao Lyu:** writing – original draft, writing – review and editing. **Jiale Chao:** writing – original draft, writing – review and editing. **Bo Fu:** conceptualization, investigation, funding acquisition, visualization, methodology.

## Data Availability

The data that support the findings of this study are available from the corresponding author upon reasonable request.
